# Evident hypopigmentation without other ocular deficits in Dutch patients with oculocutaneous albinism type 4

**DOI:** 10.1038/s41598-021-90896-y

**Published:** 2021-06-02

**Authors:** C. C. Kruijt, N. E. Schalij-Delfos, G. C. de Wit, R. J. Florijn, M. M. van Genderen

**Affiliations:** 1 Bartiméus Diagnostic Center for Complex Visual Disorders, Zeist, The Netherlands; 2 Department of Ophthalmology, Leiden University Medical Center, Leiden, The Netherlands; 3 Department of Clinical Genetics, Amsterdam University Medical Center, Location AMC, Amsterdam, the Netherlands; 4 Department of Ophthalmology, University Medical Center Utrecht, Utrecht, The Netherlands

**Keywords:** Eye diseases, Skin diseases

## Abstract

To describe the phenotype of Dutch patients with oculocutaneous albinism type 4 (OCA4), we collected data on pigmentation (skin, hair, and eyes), visual acuity (VA), nystagmus, foveal hypoplasia, chiasmal misrouting, and molecular analyses of nine Dutch OCA4 patients from the Bartiméus Diagnostic Center for complex visual disorders. All patients had severely reduced pigmentation of skin, hair, and eyes with iris transillumination over 360 degrees. Three unrelated OCA4 patients had normal VA, no nystagmus, no foveal hypoplasia, and no misrouting of the visual pathways. Six patients had poor visual acuity (0.6 to 1.0 logMAR), nystagmus, severe foveal hypoplasia and misrouting. We found two novel variants in the *SLC45A2* gene, c.310C > T; (p.Pro104Ser), and c.1368 + 3_1368 + 9del; (p.?). OCA4 patients of this Dutch cohort all had hypopigmentation of skin, hair, and iris translucency. However, patients were either severely affected with regard to visual acuity, foveal hypoplasia, and misrouting, or visually not affected at all. We describe for the first time OCA4 patients with an evident lack of pigmentation, but normal visual acuity, normal foveal development and absence of misrouting. This implies that absence of melanin does not invariably lead to foveal hypoplasia and abnormal routing of the visual pathways.

## Introduction

Oculocutaneous albinism (OCA) is characterized by hypopigmentation of skin, hair, and eyes, reduced visual acuity (VA), nystagmus, foveal hypoplasia, and misrouting of the visual pathways^1^. Oculocutaneous albinism type four (OCA4) is one of eight known non-syndromic types of albinism with autosomal recessive inheritance. To date seven genes are known causing non-syndromic OCA, OCA5 concerns a chromosomal region containing a gene yet to be identified^2^. OCA4 is caused by variants in the *SLC45A2* gene, mapped to chromosome 5p13 (OCA4; OMIM #606,574). Its protein, the membrane-associated transporter (MATP), is located in melanosomes. The exact function of MATP is unknown, but it probably plays an important role in the membrane transport of melanosomes^3^. Knockdown of MATP results in a lower pH level in the melanosomes^4^. Tyrosinase activity is inhibited by an acidic environment, and consequently variants in *SLC45A2* reduce melanin synthesis by lowering or inhibiting tyrosinase The first OCA4 patient, a Turkish patient reported in 2001 by Newton et al*.,* showed complete lack of pigmentation of skin, hair and eyes^5^. Since then, many patients have been described with variable phenotypes, from complete absence of pigmentation to subtle hypopigmentation only^1,6–30^. World prevalence of OCA4 is estimated around 1:100.000, which is 3–19% of all OCA cases^6,8,15,16,18,25,26,31–33^. In Japan OCA4 is more common, with a frequency of 27% of all OCA cases^34^. In the Netherlands, approximately 4% of OCA is caused by variants in *SLC45A2*^1^. In this study, we describe our cohort of OCA4 patients in the Netherlands, including detailed ophthalmic information.

## Patients and methods

**Figure 1:**
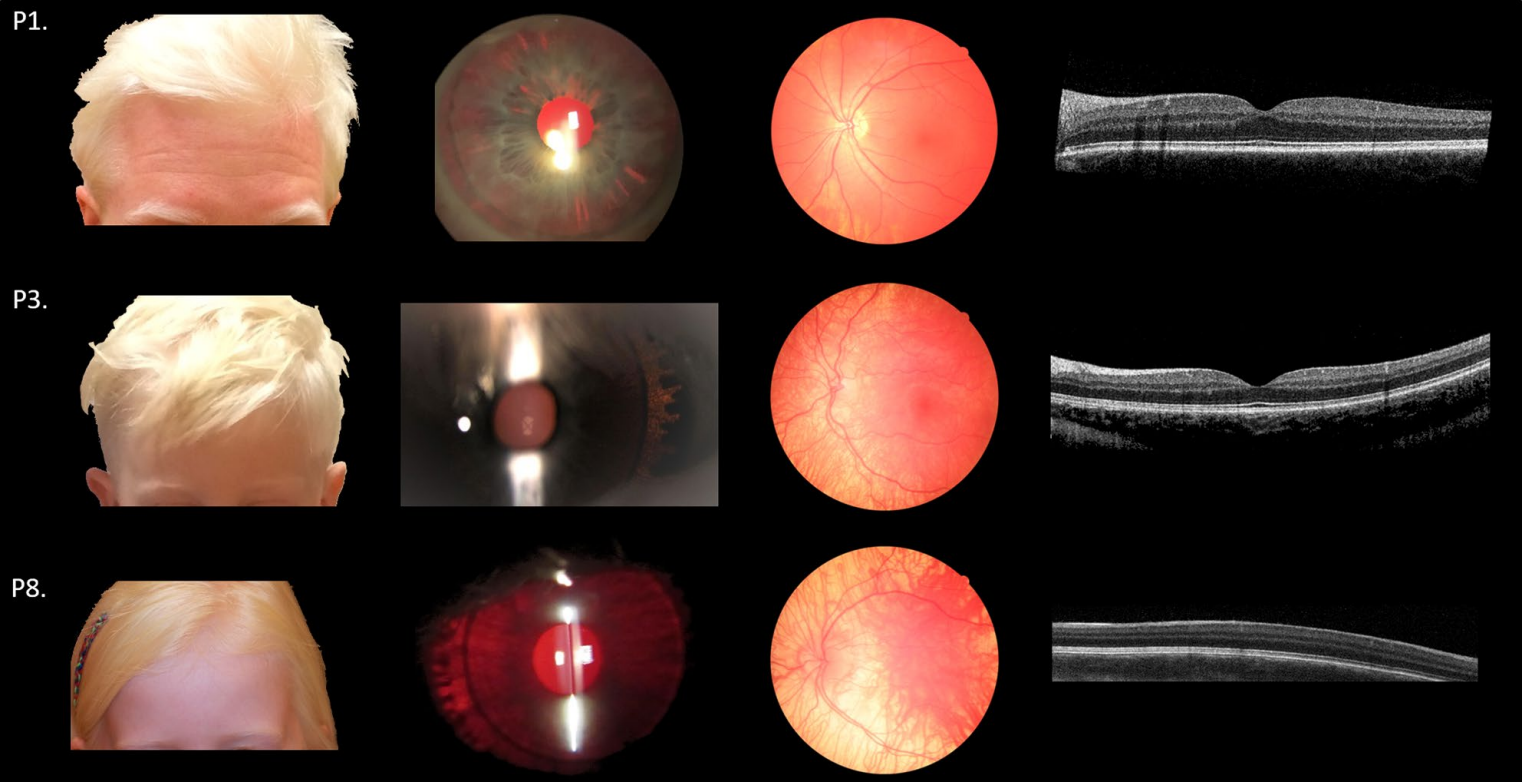
Clinical features of OCA4 patients. Pigmentation of skin and hair, iris translucency, fundus pigmentation, and optical coherence tomography of patients P1, P3, and P8. Note the complete absence of pigment of skin and hair in P1 and P3, iris translucency, fundus hypopigmentation, and completely normal foveal structure. In P8 pigmentation is comparable to P1 and P3, but no foveal pit, widening of the outer nuclear layer or elongation of the photoreceptors can be detected (foveal hypoplasia grade four according to the scheme of Thomas et al.^35^).

This study was approved by the Medical Ethics Committee of the Leiden University Medical Center and adhered to the tenets of the Declaration of Helsinki. Informed consent was obtained from all participants and/or legal guardians. An additional informed consent was obtained for the publication of the images from the patients in Fig. 1.

We identified nine patients from six families with two variants in *SLC45A2* from the databases of Bartiméus Diagnostic Center for complex visual disorders. Analyses were performed according to the ACMG guidelines by the laboratory of the Amsterdam University Medical Center, location AMC, a certified clinical laboratory. An albinism panel was screened with next-generation sequencing (NGS) for variants in the following albinism associated genes: GPR143 (OA1), TYR (OCA1), OCA2 (OCA2), TYRP1 (OCA3), SLC45A2 (OCA4), SLC24A5 (OCA6), LRMDA (OCA7), LYST (Chédiak-Higashi) and HPS1, AP3B1, HPS3, HPS4, HPS5, HPS6, DTNBP1, BLOC1S3, BLOC1S6 (Hermansky-Pudlak 1–9: respectively). Eight patients were of Dutch origin, patient 9 was of Syrian-Turkish descent. None of the patients had any interventions affecting visual acuity. We retrospectively collected data on pigmentation levels of skin, hair, and eyes, visual acuity (VA), and other ophthalmic features of albinism, -i.e. nystagmus, foveal hypoplasia, and misrouting of the optic nerve fibers. We obtained spectral-domain optical coherence tomography (OCT) scans with RT-Vue (Optovue, Fremont, CA) to assess the amount of foveal hypoplasia in all patients. To ensure we captured the foveal region we used line scans as well as MM6 scans (12 radial line scans). In patients P1, P3, and P8, we also obtained scans with Canon OCT A-1 (Kawasaki, Japan), not affecting the grade, but with a qualitatively better image. We graded the foveal hypoplasia according to the scheme of Thomas et al*.* with grade 1 and 2 not having incursion of the inner retinal layers, and grade 3 and 4 also affecting the photoreceptor differentiation^35^. Multichannel visually evoked potentials (VEPs) were recorded to determine misrouting. We used pattern onset VEPs for the assessment of misrouting in adults, and older children, and we used flash VEP for young children, according to ISCEV standards^36,37^. We used the differential signal of the electrodes on the left hemisphere minus that of the electrodes on the right hemisphere to calculate the chiasm coeffeicient. To conclude if misrouting was present we used the cutoff values calculated by Kruijt et al.^37^.

## Results

Patient data are shown in Table 1. P1, P2, and P3 were unrelated. They had very pale skin, were unable to tan, were very sensitive to sun exposure, had white to very light blond hair, white eyelashes, and blue irides, translucent over 360 degrees (grade 3–4 iris translucency^1^). P1 had grade 1 fundus hypopigmentation (only hypopigmention in the (mid)periphery^1^), but in P2, and P3 choroid vessels were easiliy visible in the posterior pole (grade 2). The main complaint of the patients was photophobia. P1 and P2 had good VA of -0.1 logMAR (1.25 Snellen). VA of P3, a four year old boy, was 0.3 logMAR (0.5 Snellen), which was within the normal range for his age. None of the three patients had nystagmus, foveal hypoplasia, or misrouting. Misrouting was absent in pattern onset as well as flash VEP in all three patients. They were diagnosed with OCA4 based on evident hypopigmentation, and two disease causing variants in the SLC45A2 gene. None of the patients carried variants in other albinism associated genes, including any of the known hypomorphic alleles in TYR and OCA2. P2 and P3 carried novel variants, c.310C > T; (p.Pro104Ser), and c.1368 + 3_1368 + 9del (p.?) respectively. (Table 1) In all three patients, segregation analysis showed that the variants were located on different alleles. Iris transillumination, fundus images, and OCT-scans of P1 and P3 are illustrated in Fig. 1.

**Table 1:** Clinical and molecular findings.

Subject age/gender	Variants SLC45A2 (Chr5: NM_016180.4)	Pigmentation skin and hair	*VA	Refractive error	Nystagmus	Iris translucency	Fundus pigmentation^a^	Foveal hypoplasia^b^	Misrouting
P1 31/male	c.1502C > A (p.Ala501Asp) c.1567G > A (p.Ala523Thr)	White eyelashes, white hair, pale skin	− 0.1	+ 0.50D/ − 2.50 × 160 + 0.25D/ − 2.00 × 28	No	Grade 4	Grade 1	No hypoplasia	No
P2 18/female	c.125 T > C (pMet42Thr) c.310C > T (p.Pro104Ser)	Blond eyelashes, very light blond hair, pale skin	− 0.1	+ 3.00D/ − 0.50 × 167 + 3.00D/ − 0.50 × 34	No	Grade 3	Grade 2	No hypoplasia	No
P3 4/male	c.1082 T > C (p.Leu361Pro) c.1368 + 3_1368 + 9del (p.?)	White eyelashes, white hair, pale skin	0.3	+ 4.50D/ − 0.25 × 180 + 4.50D/ − 0.25 × 180	No	Grade 3	Grade 2	No hypoplasia	No
P4 31/female	c.533_534dup (p.Gly179Argfs*23) c.1082 T > C (p.Leu361Pro)	White eyelashes, white hair, pale skin	0.6	+ 4.00D/ − 4.75 × 11 + 1.75D/ − 3.00 × 180	Yes	Grade 4	Grade 2	Grade 4	Yes
P5 48/female	c.533_534dup (p.Gly179Argfs*23) c.1082 T > C (p.Leu361Pro)	White eyelashes, white hair, pale skin	1.0	+ 0.50D/ − 4.50 × 98 + 1.00D/ − 3.00 × 83	Yes	Grade 4	Grade 2	Grade 4	Yes
P6 12/male	Homozygous c.264del (p.Gly89Aspfs*24)	White eyelashes, white hair, pale skin	0.9	+ 3.50D/ − 2.75 × 180 + 5.00D/ − 2.00 × 176	Yes	Grade 4	Grade 3	Grade 4	Yes
P7 12/male	Homozygous c.264del (p.Gly89Aspfs*24)	White eyelashes, white hair, pale skin	0.7	+ 4.00D/ − 2.50 × 169 + 3.25D/ − 2.50 × 164	Yes	Grade 4	Grade 3	Grade 4	Yes
P8 6/female	Homozygous c.264del (p.Gly89Aspfs*24)	White eyelashes, white hair, pale skin	0.8	+ 3.25D/ − 2.75 × 178 + 5.50D/ − 3.50 × 8	Yes	Grade 4	Grade 3	Grade 4	Yes
P9 5/male	Homozygous c.277G > A (p.Asp93Asn)	Blond eyelashes, very light blond hair, pale skin	0.6	+ 2.25D/ − 1.75 × 10 + 3.00D/ − 1.50 × 175	Yes	Grade 4	Hypopigmentation, no fundus image for grading	Hypoplasia, no OCT for grading	Yes

*Visual acuity in logMAR, ^a^According to the grading of Kruijt et al.^1^, ^b^According to the grading of Thomas et al*.*^35^.

**Figure 2:**
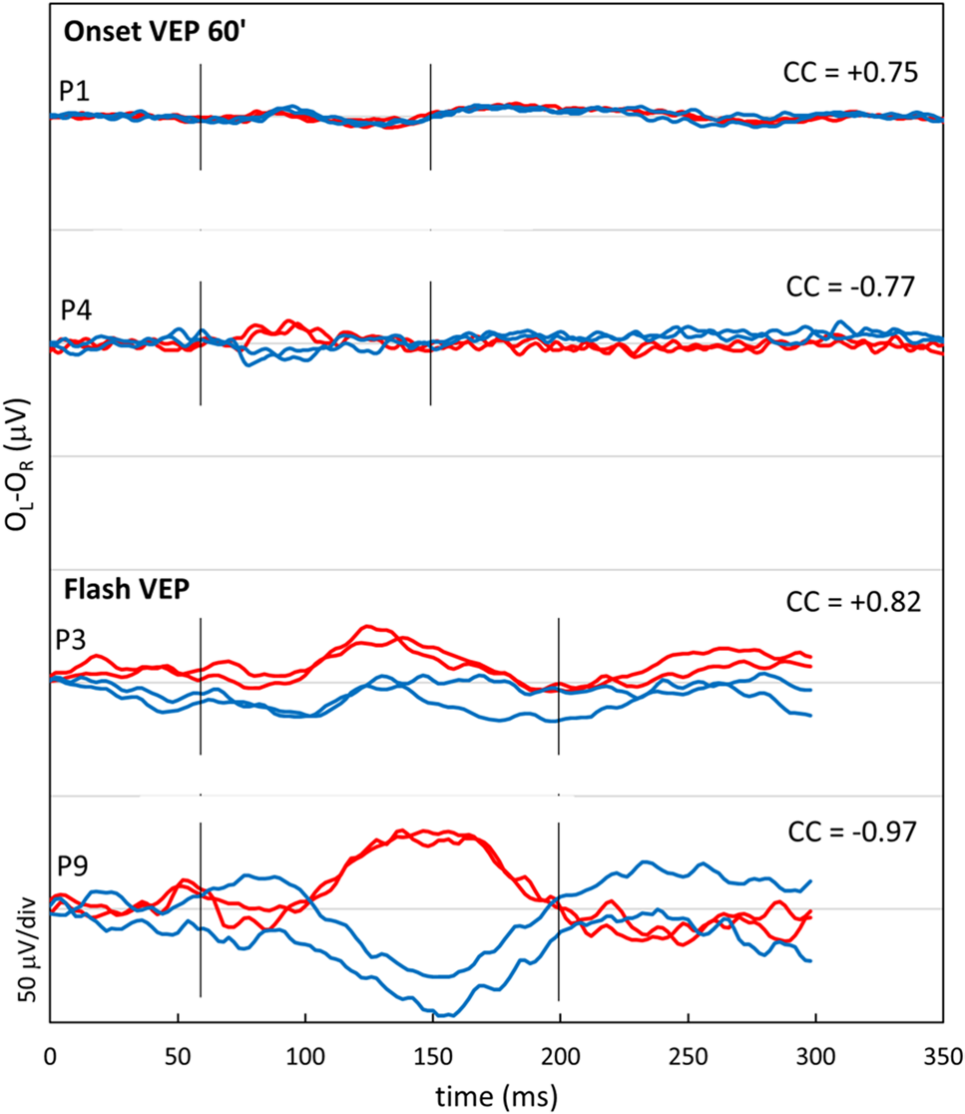
Visually evoked potentials. Difference signal of the pattern onset and flash visually evoked potentials (VEP) between the electrode on left hemisphere and right hemisphere recorded twice from the right eye (red lines) and left eye (blue lines). Based on these interhemispheric difference signals a chiasm coefficient was calculated over 60–150 ms (ms) for the pattern onset and the 60–200 ms for the flash VEP. For adults the pattern onset was used to detect misrouting, and for children the flash VEP^37^. P1 and P4 were both 31 years of age. The signals in P1 are symmetrical, while misrouting is detected in P4. P3 was two years of age during VEP test, and has symmetrical responses. VEP test of P9, tested at the age of eight months old, has a negative chiasm coefficient (cc) of -0.98, proving misrouting.

P4 and P5 were sisters, P6 and P7 twin brothers. P9 did not have siblings with OCA4. P4–P9 also showed severe lack of pigmentation of skin, hair, and eyes, comparable to patients P1, P2, and P3. But, in contrast, visual function was severely affected. They all had poor VA (0.6 to 1.0 logMAR), nystagmus, severe foveal hypoplasia (grade four^35^), and misrouting. (Table1, and Fig. 1). Examples of the results of pattern onset and flash VEP-testing are shown in Fig. 2 (P1 and P4 pattern onset, and P3 and P9 flash VEP).

## Discussion

In this report, we describe for the first time three unrelated Dutch OCA4 patients, with evident hypopigmentation of skin, hair, and iris, but normal visual acuity, foveal development and routing of the visual pathways. Both missense variants found in P1, c.1502C > A; p.Ala501Asp and c.1567G > A; p.Ala523Thr, are localized in the major facilitator superfamily domain. c.1502C > A. The p.Ala501Asp variant was previously reported in two heterozygous albinism patients, one was severely affected (not specified) and the other patient had mild hypopigmentation and nystagmus (further details missing)^8,25^. The second variant, c.1567G > A; p.Ala523Thr, was described in one compound heterozygous albinism patient without phenotypic description^38^. The missense variants c.125 T > C and c.310C > T found in P2 predict the amino acid substitution p.Met42Thr and p.Pro104Ser respectively, changing a highly conserved amino acid. The second variant c.310C > T; p.Pro104Ser was novel, and our laboratory classified it as likely pathogenic, Grantham distance (74), AGVGD C65, SIFT Deleterious, Polyphen probably damaging. P3 had one previously reported variant, c.1082 T > C; p.Leu361Pro, and one novel variant, c.1368 + 3_1368 + 9del p.?. The missense variant c.1082 T > C; p.Leu361Pro has been reported in a homozygous patient that was severely affected in melanin synthesis and visual function^26^. The variant was also found in severely affected P4 and P5 from our series. The novel variant c.1368 + 3_1368 + 9del p.? probably results in abnormal splicing of the *SLC45A2* RNA, causing a frameshift by skipping exon 6 according to three out of four prediction programs. Prediction programs scores were: SpliceSitefinder wildtype (WT) 76.6, variant 14.7; MaxEntScan WT 5.4, variant 3.3; NNSPLICE WT 0.6, variant no prediction; Genesplicer WT no predicition, and variant no prediction. The pathological significance of the novel variants has to be further investigated, for example RNA analysis to confirm the effects on exon 6 from the prediction programs. For c.310C > T; p.Pro104Ser functional analysis may be performed as described by Konno et al*.*^39^.

Unravelling the genotypic profile of all these cases does not provide a decisive clue for the variety in phenotypic presentation in this cohort. It is remarkable, that in our series of nine OCA4 patients, all patients showed obvious lack of pigmentation of skin, hair, and eyes. But, concerning visual function and ocular development, they were either on the poorer end of the spectrum for albinism, or were not affected at all. Even though visual function was not affected in P1, P2, and P3, sufficient diagnostic criteria for albinism proposed by Kruijt et al*.* were met, i.e. a molecular diagnosis, combined with a major criterion (iris translucency) and at least two minor criteria ( hypopigmentation of skin, hair, and fundus (all minor criteria))^40^.

Since the first patient reported in 2001, many patients with OCA4 have been described. In contrast to the homogeneous hypopigmentation phenotype we found in our cohort, in the literature the degree of pigmentation seems to vary from complete lack of pigmentation to very mild hypopigmentation^1,6–30^. While most reports describe pigmentation levels of skin and hair, ophthalmic details are usually scarce. Some studies report absence of nystagmus in some patients^9,10,13,15,17–20^. Visual acuities in OCA4 patients in earlier reports ranged from  − 0.1 to 1.5 logMAR, with most patients having poor VA^6,9,14,16,18,25,26,28,41^. Only three patients were described with normal VA, and all three had only mild or no hypopigmentation^9,18,26^. Rundshagen et al*.* described a patient with VA of 0.2 logMAR, subtle hypopigmentation, and nystagmus^26^. The second patient with good VA was of Japanese origin. His VA was 0.0 logMAR, he had brown hair and mild hypopigmentation of the skin, with the ability to tan. Other ophthalmic details were missing^9^. An Italian patient with good VA (< 0.2 logMAR), no nystagmus, minimal iris translucency, and no fundal hypopigmentation is the only OCA4 patient decribed, to our knowledge, without foveal hypoplasia. In this patient misrouting was present, and molecular analysis revealed two variants in the *SLC45A2* gene, c.619C4G (p.L207V) and c.606G4C (p.W202C)^18^. Until now, no patients have been described with evident lack of pigmentation of skin, hair, and eyes, but with normal ocular development, -i.e. normal VA, no nystagmus, no foveal hypoplasia and normal routing of the optic nerve fibers. Especially the absence of foveal hypoplasia is remarkable, occurring in less than 0.7% of albinism patients^1^.

It is still unclear why variants in genes responsible for melanin synthesis cause defects in the development of the visual system. It is assumed that lack of melanin in the retinal pigment epithelium is responsible for excessive crossing of the optic nerve fibers and foveal hypoplasia. Pigment epithelium-derived factor (PEDF) is a negative regulator of angiogenesis and plays an important role in the formation of the foveal avascular zone^42^. PEDF is decreased in the absence of tyrosinase, and therefore foveal hypoplasia could be caused by reduced PEDF^43,44^. However, a role for PEDF in chiasmal misrouting has not been established.

Generally, albinism patients with more severe hypopigmentation have more severe foveal hypoplasia and worse visual acuity^1^. Patients P4–P9 from this report conform to this phenotype. In contrast, P1, P2, and P3 demonstrate that normal foveal development, and normal routing of the optic nerve fibers can occur despite an evident lack of melanin. Non pathogenic variants in *SLC45A5* may cause lightly pigmented skin and hair without an ocular phenotype. However, the variants found in P1, P2, and P3 were likely pathogenic, and importantly, the patients’ hypopigmented phenotype was not restricted to skin and hair, as they did have grade 3–4 iris translucency.

The OCA4 phenotype of patients P1, P2, and P3 seems the very opposite of the phenotype of the FHONDA syndrome, caused by variants in *SLC38A8*^45–48^. Patients with FHONDA have nystagmus, poor VA, severe foveal hypoplasia and misrouting, but no pigmentation defect. The FHONDA syndrome provided the first convincing evidence that lack of melanin is not the only determining factor in the combined occurrence of foveal hypoplasia and misrouting^45^.

The three OCA4 patients with normal visual development we describe in this report, are further proof that the relationship between pigmentation defect and ocular deficits in albinism is more complicated than previously thought. Further research is needed to unravel the mechanisms that cause some OCA4 patients to have a severe albinism phenotype, while others do not show any ocular deficit, apart from iris translucency.

## Data Availability

All data that are not included in this published article are available from the corresponding author on request.
